# Discharge interventions for older patients leaving hospital: protocol for a systematic meta-review

**DOI:** 10.1186/s13643-016-0222-8

**Published:** 2016-03-16

**Authors:** Elaine O’Connell Francischetto, Sarah Damery, Sarah Davies, Gill Combes

**Affiliations:** NIHR CLAHRC West Midlands - Theme 4 Chronic Diseases, School of Health & Population Sciences, University of Birmingham, Edgbaston, Birmingham, B15 2TT UK; Behavioural Brain Sciences Unit, Institute of Child Health, University College London, 30 Guilford Street, London, WC1N 1EH UK

**Keywords:** Hospital discharge, Older people, Meta-review, Review of reviews

## Abstract

**Background:**

There is an increased need for additional care and support services for the elderly population. It is important to identify what support older people need once they are discharged from hospital and to ensure continuity of care. There is a large evidence base focusing on enhanced discharge services and their impact on patients. The services show some potential benefits, but there are inconsistent findings across reviews. Furthermore, it is unclear what elements of enhanced discharge interventions could be most beneficial to older people. This meta-review aims to identify existing systematic reviews of discharge interventions for older people, identify potentially effective elements of enhanced discharge services for this patient group and identify areas where further work may still be needed.

**Methods/design:**

The search will aim to identify English language systematic reviews that have assessed the effectiveness of discharge interventions for older people. The following databases will be searched: Medline, Embase, PsycINFO, HMIC, Social Policy and Practice, CINAHL, the Cochrane Library, ASSIA, Social Science Citation Index and the Grey Literature Report. The search strategy will comprise the keywords ‘systematic reviews’, ‘older people’ and ‘discharge’. Discharge interventions must aim to support older patients before, during and/or after discharge from hospital. Outcomes of interest will include mortality, readmissions, length of hospital stay, patient health status, patient and carer satisfaction and staff views. Abstract, title and full text screening will be conducted independently by two reviewers. Data extracted from reviews will include review characteristics, patient population, review quality score, outcome measures and review findings, and a narrative synthesis will be conducted.

**Discussion:**

This review will identify existing reviews of discharge interventions and appraise how these interventions can impact outcomes in older people such as readmissions, health status, length of hospital stay and mortality. The review could inform practice and will help identify where further research is needed.

**Systematic review registration:**

PROSPERO CRD42015025737.

**Electronic supplementary material:**

The online version of this article (doi:10.1186/s13643-016-0222-8) contains supplementary material, which is available to authorized users.

## Background

There has been a recent movement in healthcare policy and practice towards providing additional patient-centred care (e.g. enhanced hospital discharge services, multidisciplinary working or home care), to support the needs of the growing population of older patients (people aged over 60) [1, 2]. Suboptimal care of older people has been identified as a significant issue, and there is a renewed focus on collaboration and effective communication between secondary, primary and social care partners to improve care services for the older patient [1, 3–5].

The older populations are more likely than younger individuals to suffer from multiple morbidities and problems such as frailty [4, 6]. Conditions associated with the older population can cause people to lose weight, have worsened mobility and increased risk of falls, as well as being susceptible to other health problems [4, 6]. This means the older population can take longer to recover from illness and require more continuity of care [6]. Hospital admissions in the elderly population continue to increase, and support for older people is particularly important around the time of discharge, for example home assessments prior to discharge, a geriatric assessment during discharge or multidisciplinary home care after discharge [1, 7]. Discharge interventions can be broadly defined as an intervention, which intends to revise the discharge process for patients being discharged from hospital care [8]. Discharge interventions can occur at different points in the care pathway; for example, comprehensive geriatric assessment and individual care plans can be completed in hospital to respond to the needs of older patients once they are discharged from hospital [6, 9]. Assessment tools and care plans can identify what support older patients need after discharge, and discharge interventions are well placed to help inform appropriate care pathways for the more elderly population across a number of settings.

Primary research on discharge interventions has led to a substantial number of systematic reviews being conducted in this area, but they have varied in their focus. Some reviews have not focused on a specific population [10–12] whilst others have targeted different patient groups such as stroke patients [13], patients with multiple morbidities [14] and older patients [9, 15–20]. Some reviews have included different types of discharge interventions such as early supported discharge [12, 13], geriatric assessment [9, 15], home care [10, 19], medical day care [16] and multidisciplinary care teams [17]. In addition to differences in the nature of the interventions focused on, these reviews have also varied in terms of the healthcare partners and staff involved, intervention duration, time points for data collection and the healthcare setting of support delivery [11, 14, 16, 17, 20].

There is evidence to suggest that in some contexts, discharge interventions can reduce length of hospital stay [10, 12]; reduce readmissions [10]; reduce admittance to residential care/institutions [9, 12, 13]; improve clinical outcomes [9, 13] and increase patient satisfaction [12, 13], independence and mobility [10, 12, 13]. However, the results of systematic reviews have been conflicting, and systematic reviews have identified variability between primary studies [9–12, 14, 17]. For example, Shepperd et al. [12] found that ‘hospital at home’ interventions can increase readmissions for older patients but that there were no significant differences in readmissions for stroke patients. In contrast, Shepperd et al. [10] found that ‘discharge planning’ interventions can reduce readmissions in older patients. This highlights the uncertainty regarding whether discharge interventions can improve outcomes or whether there are specific elements of discharge interventions that are effective for certain populations. It is still unclear what impact a discharge intervention has on post-discharge health and social care usage; costs (patient, health, community and social care); patient-reported outcomes; functional status and carers’ wellbeing and views (such as intervention acceptability) [11–13, 16]. Therefore, despite a large evidence base for systematic reviews of different types of discharge interventions, there is continuing uncertainty regarding the specific aspects of discharge interventions that are most effective and who they are beneficial for.

It would be useful to identify what discharge interventions there are specifically for the older population in systematic review evidence, as there are numerous discharge interventions available for this group. These may include assessing the rehabilitation needs of patients, planning and delivering specific support in particular settings to patients after they are discharged (such as physiotherapy in the community or falls assessment in the patient’s home) [10, 12, 21]. This body of research on discharge interventions has meant that interventions are now implemented into practice for certain patient groups; however, it is still unclear what best practice is for discharge interventions [22, 23].

Meta-reviews are useful when systematic reviews have already been conducted in the research area of interest, as they can provide new insights into the existing evidence base [24]. A meta-review is a systematic review of systematic reviews, which can be used to summarise the data from systematic reviews conducted on the same subject; it can also be referred to as an overview of reviews or umbrella review [25, 26]. A meta-review of discharge interventions was conducted in 2007 [11], but this review did not focus on older people, so an up to date and more focused meta-review is overdue. The purpose of this meta-review is to firstly identify existing systematic reviews of discharge interventions for older people, secondly identify potentially effective elements of discharge services for this patient group, and lastly to identify where further work may still be needed.

### Objectives

1. Identify reviews of different discharge interventions which aim to support older patients.
2. Identify the characteristics of the different subgroups of older patients included in these reviews.
3. Identify any interventions or specific elements of interventions which have been effective in achieving clinical, patient, staff and carer outcomes:
  - Patient mortality, health status, quality of life, dependency (using activities of daily living scores) and satisfaction.
  - Patient hospital length of stay and readmission rates.
  - Carer outcomes including acceptability of intervention, satisfaction, burden and quality of life.
  - Staff outcomes including views on service and workload.
  - Costs and resource use.
4. Identify which patient-reported outcome measures have been used to measure outcomes in older patients.
5. Identify areas where further research is needed.

## Methods/design

The protocol for this meta-review has been written in accordance with the PRISMA-P (Preferred Reporting Items for Systematic Review and Meta-Analysis Protocols) guidelines [27] and has been registered with PROSPERO (registration number CRD42015025737).

### Study design

Only systematic reviews, meta-analysis or other systematic meta-reviews will be included in this meta-review. Eligible reviews must have the following key characteristics as defined by Higgins and Green [24]:

- A clearly stated set of objectives with pre-defined eligibility criteria for studies.
- An explicit, reproducible methodology.
- A systematic search that attempts to identify all studies that would meet the eligibility criteria.
- An assessment of the validity of the findings of the included studies, for example through the assessment of risk of bias.
- A systematic presentation, and synthesis, of the characteristics and findings of the included studies.

### Population

The population of interest for this review is older patients (i.e. those over 60 years of age) [28]. Where older people are not exclusively included, a review is still eligible for inclusion if stand-alone data can be extracted on the patient group of interest.

### Interventions

Systematic reviews that focus on interventions that provide additional support or adapt the process around the time of discharge for older patients who are a hospital inpatient. This could be an intervention before, during and/or after discharge from hospital. A previous systematic review [8] developed categories for discharge interventions whilst undertaking their systematic review of discharge arrangements for older people. The categories defined by Parker et al. will be used in this review as they are informed by the evidence base and are used in a previous meta-review [8, 11]. The categories for discharge interventions defined by Parker et al. [8 pg 9] are as follows:

- Discharge planning schemes: primarily interventions that utilise comprehensive discharge planning protocols.
- Discharge support schemes: a variety of models in which new and existing services are targeted at recently discharged patients, including schemes with early discharge from inpatient hospital care.
- Geriatric assessment programmes: assessment services focused on hospital inpatients and patients recently discharged from hospital.
- Educational programmes: a fairly distinct group of studies with objectives of educating patients in aspects of management of their illness.

These categories are not mutually exclusive, but they will provide structure to the review and analysis [8]. Any post-discharge setting for older patients will be included. Settings may include a patient’s own home, a carer/family member’s home, community rehabilitation settings or residential nursing home.

### Comparators

Due to hospitals offering different types of discharge services and support, comparators of any type of standard care or alternative interventions will be included in this review. Where possible, the services used as comparators will be summarised and then grouped to allow comparison.

### Outcomes

Outcome measures of interest will include (but are not restricted to) the following:

- Mortality
- Readmissions (all cause and condition specific)
- Length of hospital stay
- Patient health status (including patient-reported health status, quality of life, functional outcomes and psychological outcomes)
- Patient satisfaction
- Admission to institutional care
- Carer outcomes (including health status, service preference, burden and satisfaction)
- Staff outcomes (including staff views and workload)
- Care process measures (e.g. number of complications)
- Costs and resource use (including outpatient use, social care use, primary care use and emergency department visits)

### Exclusion criteria

- Interventions that focus solely on populations that cannot be classified as older people and where there is no stand-alone data that can be extracted on the population of interest, e.g. a review focusing on post-pregnancy discharge services.
- Interventions which do not aim to support patients around the time of discharge from hospital.
- Specialist mental health discharge services except discharge services for delirium or dementia. These services will be included as these conditions are common in the older population [6].
- Reviews where there is no full paper and/or results such as abstract only citations from conference proceedings or published protocols of systematic reviews.
- Reviews written in languages other than English (there is no restriction regarding the language of source data included in the reviews).
- Reviews focusing solely on less economically developed countries (LEDCs). Interventions in LEDCs will not be comparable due to different standards of living, quality of medical care, life expectancies and quality of life. If there is uncertainty regarding whether a country is a more or less economically developed country, the United Nations country classification document will be used [29].
- Interventions evaluating the effectiveness of drugs administered to patients.
- Reviews scoring less than 5 on the AMSTAR (a measurement tool to assess systematic reviews) checklist (see ‘Assessment of review quality’ section).

### Literature search strategy

This meta-review will have no restriction on review publication date. Due to pragmatic reasons, the search has been limited to reviews written in English, which could introduce a language bias [24].

The following databases will be searched from inception:

- Medline (using Ovid)
- Embase (using Ovid)
- PsycINFO (using Ovid)
- Health Management Information Consortium (HMIC) (using Ovid)
- Social Policy and Practice (using Ovid) (database includes Social Care Online and AgeInfo)
- Cumulative Index to Nursing and Allied Health Literature (CINAHL)
- Cochrane Library (includes Database of Abstracts of Reviews of Effects (DARE))
- Applied Social Sciences Index and Abstracts (ASSIA)
- Social Science Citation Index
- Grey Literature Report (www.greylit.org)

To ensure that systematic reviews in non-journal sources and within the grey literature are also captured, the search will include databases which are sources of grey literature. The reference lists of each included review will be screened for relevant reviews. Key terms such as ‘discharge services for the elderly review’ will be put into the Google search engine to identify any additional grey literature or published reviews, which may not be indexed in the databases above. Search results in Google are extensive and ordered by relevance; therefore, the screening of Google will not be systematic and will be stopped once search results are no longer deemed relevant by the review team.

### Search terms

Searches will be developed and combined using broad search terms, key words, Medical Subject Headings (MESH) and filters for ‘systematic reviews’, ‘older people’ and ‘discharge’.

Data are available on the effectiveness of different filters to identify systematic reviews, and previous studies have conducted reviews of the best search strategies for identifying systematic reviews in databases (such as Medline, Embase and CINAHL) [30, 31]. Where available, the systematic review search filter that maximises specificity will be used for each database. This will give the greatest likelihood that potentially relevant articles will be identified and non-relevant articles will be omitted from the search results [30, 31]. If multiple search filters have similar rates of specificity, then further data such as sensitivity and ‘number needed to read’ will be assessed before deciding which strategy to use. When a systematic review search filter is not available for a database, the search will be based on the Medline search strategy and key search terms will be used to identify systematic reviews, meta-reviews and meta-analyses.

The preliminary search strategy for Medline is provided in Additional file 1. This will be modified accordingly for each additional database. Once the search strategies are finalised, these will be uploaded to PROSPERO.

### Study selection and screening

First, all of the citations will be transferred to Refworks and duplicates will be removed. Citations will then be exported into Microsoft Excel to check for any remaining duplicates. The titles and abstracts of all citations will be independently screened for eligibility against the inclusion and exclusion criteria by two reviewers. A review will be taken forward to the full text screening stage if (please see full decision algorithm in Table 1):

**Table 1:** Decision algorithm after abstract and title screening

Screening decision reviewer 1	Screening decision reviewer 2	Decision
Eligible	Eligible	Full text screening
Eligible	Unsure	Full text screening
Unsure	Eligible	Full text screening
Unsure	Unsure	Full text screening
Not eligible	Unsure	Full text screening
Unsure	Not eligible	Full text screening
Not eligible	Not eligible	Excluded

1. The study is considered eligible by both of the reviewers.
2. The reviewer is unsure of a review’s eligibility and the full text needs to be reviewed.
3. There is disagreement between the two reviewers as to whether the study is relevant.

Initial title and abstract screening will be undertaken using Microsoft Excel. All inclusion/exclusion decisions will be documented: this will allow the results of the first stage of screening to be reviewed. After title and abstract screening, the full texts of potentially eligible papers will be obtained and screened (again by two independent reviewers). If the two reviewers do not agree about inclusion of a review, they will meet to discuss it. If agreement regarding inclusion/exclusion cannot be reached, the review will be discussed by the wider research team who will then make a decision. Results of the search and screening process will be reported in a PRISMA flow diagram. The above screening process will be piloted on the first 100 results of the Ovid Medline search, and this will allow the process to be refined if necessary before completing the whole review. Cohen’s kappa index of inter-rater reliability will be used to assess agreement between the two reviewers. A score between 0.61 and 1.0 will be considered acceptable as anything above 0.61 is defined as substantial or almost perfect agreement [32]^.^

### Data extraction and management

Data extraction will be completed independently by the two reviewers. Any disagreements will be discussed, and if agreement cannot be reached, they will be discussed by the wider research team. Extracted data will be collated in word documents, and possible answers with tick boxes will be used where possible. Please see below details of what the data extraction form will include and brief examples of tick boxes we will have:

1. Identifying features
  - Reference ID number
  - Citation
  - Country of publication
2. Review characteristics
  - Type of review (for example systematic review or meta-review)
  - Which category does the review fit into as defined by Parker et al.?
    Discharge planning schemes
    Discharge support schemes
    Geriatric assessment programmes
    Educational programmes
  - Databases searched (for example Medline, Embase, PubMed or The Cochrane Library)
  - Additional sources (for example reference lists or contacting experts in field)
  - Years searched
  - Geographical scope (for example no restriction, USA or EUROPE)
  - Language restrictions of included studies (for example English)
  - Healthcare settings included in review (for example general practice surgeries, hospitals or community group/service)
  - Overall aim of the review
  - Review research questions/objectives
  - Study designs included (for example randomised controlled trials (RCTs), controlled before and after studies or cohort studies)
  - Number of studies included in review
  - Number of published articles included in review
  - Data synthesis (for example meta-analysis or narrative)
  - AMSTAR score (low quality, moderate quality or high quality)
3. Review participants
  - Patient population included in review
  - Patient age range
  - Total number of participants included
4. Interventions
  - General description of intervention(s)
  - General description of comparator(s)
  - Evidence of theories or conceptual frameworks informing the intervention
  - Did intervention involve different care provided by different staff, in different settings or at different time points? (Yes or no. If yes, we will ask for specific details)
  - Where was the intervention(s) administered? (for example general practice surgeries, hospitals or community setting)
  - Staff involved in intervention(s) (for example nurse, occupational therapist or physiotherapist)
  - Timescales over which interventions were administered
  - Do interventions have a ‘handover’ back into routine care or other service? (Yes or no. If yes, we will ask for specific details)
5. Outcomes measured
  - All primary outcome
  - All secondary outcome(s)
6. Findings
  - Results of outcomes of interest (review outcomes of interest listed)
    We will collect data on each outcome regarding whether any of the following has had an impact on each outcome:
    Intervention setting
    Intervention components
    Intervention comparator
    Intervention population
    Intervention providers
    Intervention resources
  - Review conclusion(s)
  - Patient reported outcome measures that are identified in this review.
  - Review authors’ assessment of overall quality of the evidence reported in the review
  - Results of any relevant subgroup analysis
  - Any barriers and/or facilitators for implementing the interventions
    Detailed in results section
    Detailed in discussion section
  - Review conclusion(s)
  - Further research/work or gaps identified in review
    If none, consider the Population, Intervention, Comparison, Outcomes and Setting (PICOS) detailed in the systematic review question and whether these are represented in the results [33].

### Assessment of review quality

AMSTAR will be used to assess the quality of the systematic reviews, and the tool consists of an 11-item checklist [34]. Systematic reviews will be scored from 0 to 11 using the AMSTAR checklist. Previous studies reviewing the quality of systematic reviews have classified AMSTAR scores into the following groups: a low quality score ranging from 0 to 4; a moderate quality score ranging from 5 to 8 and a high quality score ranging from 9 to 11 [35, 36]. Reviews with a moderate score (five or above) will be included in this review.

### Risk of publication bias

The search strategy for this meta-review has been designed to be comprehensive and includes sources of grey literature. It is accepted that there may be some publication bias in this meta-review due to only including reviews published in English.

The meta-review will include moderate to high quality systematic reviews, and the AMSTAR checklist includes questions on publication bias assessment, whether the literature search was comprehensive and whether status of publication was used as an inclusion criteria. The scores of the AMSTAR checklist will be reviewed to assess if included reviews have scored well regarding publication bias, and this data will be reported.

### Data synthesis and analysis

A number of previous systematic reviews have faced challenges when comparing study results such as missing information [13, 16], differences in healthcare and funding structures across different nations and the date that research was conducted [10, 12, 16]. Other issues can include the difficulty of comparing specific intervention components across complex trials [10, 20]; interventions in different settings/populations, interventions with different lengths of follow-up [9, 37] and issues related to multiple publications reporting different study outcomes [38]. The issue of discordant reviews is not new, and there is guidance available to deal with this type of data. When interpreting discordant reviews, it is important to consider which review is most relevant to the meta-review research question and the quality of the reviews. A decision algorithm developed by Jadad et al. [37] will be used to assist with interpreting data from discordant reviews.

Due to the expected heterogeneity in intervention designs and characteristics, a more narrative synthesis will be performed. Included reviews will be categorised, analysed and presented according to the Parker et al. [8] categories for discharge interventions detailed in the inclusion criteria. However, there may be a need to further classify the included reviews into subcategories based on their specific characteristics; this will aid data interpretation and review comparison. Previous overview/meta-reviews in similar topic areas have used intervention characteristics and outcome measures to classify studies, but it will not be clear if this is the best approach until the review is completed [11, 39]. A clear descriptive summary and summary tables of the included studies will be produced. This will include the Cochrane handbook templates for the ‘Characteristics of included studies’ table and ‘Overview of reviews’ table [25]. If evidence is available on theories or conceptual frameworks informing the intervention, these will be reported. The main conclusions will be highlighted to correspond with the review objectives. An iterative approach will be used throughout the review process to develop the best approach for presenting the findings alongside the Cochrane templates. To provide a summary of the effective elements of discharge services, a table will be produced for each of the outcomes of interest. The tables will summarise the impact of intervention settings; intervention components; intervention providers; intervention comparators; intervention population and intervention resources on each outcome of interest (please see Table 2). When retrospectively reviewing systematic reviews for gaps, data extraction should be restricted to direct statements presented by the review authors [33]. Therefore, we plan to summarise gaps identified by the authors of the systematic reviews; if no gaps are summarised, we will use the PICOS detailed in the systematic review research question to consider whether these are represented in the results [33].

**Table 2:** Effective elements of discharge services (to be completed for each outcome of interest)

Categories of effective elements						
Intervention settings	Intervention components	Intervention providers	Intervention resources	Intervention comparator	Intervention population	Other
E.g. hospital/home	E.g. physiotherapy/geriatric assessment.	E.g. nurse/social worker	E.g. personalised advice/telephone support	E.g. standard care/alternative intervention	E.g. 60+/stroke patient	Please detail as necessary

### Peer review and patient and public involvement

In addition to discussing the study protocol with the study team, the National Institute for Health Research (NIHR) Collaboration for Leadership in Applied Health Research and Care (CLAHRC) West Midlands Chronic Disease theme patient group have been consulted to discuss the review. The patient group will also be consulted to discuss the interpretation of the findings and draft paper and to incorporate what they consider relevant to patient and public audiences [40]. Furthermore, the draft protocol will be sent for peer review through NIHR CLAHRC West Midlands. The final draft will be approved by the whole study team prior to being published.

### Ethics and dissemination

There are not considered to be any ethical concerns regarding undertaking this meta-review. Study findings will be disseminated and published. The findings from the work will inform ongoing and future work which is being undertaken by the NIHR CLAHRC West Midlands Chronic Disease Theme. As well as academic publications, the findings of the review will be disseminated to hospital teams in the West Midlands where discharge interventions for older people are part of hospital services or are being planned.

## Discussion

The large number of existing reviews on this subject has made the evidence base challenging to interpret, and it would benefit from being systematically appraised. Systematic reviews of RCTs are considered the highest level of evidence available [41]. This review has been designed to identify existing reviews of discharge intervention and detail how these interventions are structured and how they can potentially impact on outcomes relevant to older people such as readmissions, health status, length of hospital stay and mortality. Furthermore, this review will help identify gaps in the evidence base and where further research is needed.

## Additional files

Additional file 1: Medline search strategy. (PDF 354 KB)

## Abbreviations

AMSTAR: a measurement tool to assess systematic reviews
ASSIA: Applied Social Sciences Index and Abstracts
CINAHL: Cumulative Index to Nursing and Allied Health Literature
CLAHRC: Collaboration for Leadership in Applied Health Research and Care
DARE: Database of Abstracts of Reviews of Effects
HMIC: Health Management Information Consortium
MESH: Medical Subject Headings
NHS: National Health Service
NIHR: National Institute for Health Research
PICOS: Population, Intervention, Comparison, Outcomes and Setting
PRISMA-P: Preferred Reporting Items for Systematic Review and Meta-Analysis protocols
RCT: randomised controlled trial
