# Supplementary material for: Discharge interventions for older patients leaving hospital: protocol for a systematic meta-review
Source: Syst Rev. 2016 Mar 16;5:46. doi: 10.1186/s13643-016-0222-8 (PMC4793488; doi:10.1186/s13643-016-0222-8)
Supplement: Additional file 1: — Medline search strategy. (PDF 354 KB) [file 13643_2016_222_MOESM1_ESM.pdf]

Additional file 1

Medline search:

Review search

1. (review or review, tutorial or review, academic).pt.
2. (medline or medlars or embase or pubmed or cochrane).tw,sh.
3. (scisearch or psychinfo or psycinfo).tw,sh.
4. (psychlit or psyclit).tw,sh.
5. cinahl.tw,sh.
6. ((hand adj2 search$) or (manual$ adj2 search$)).tw,sh.
7. (electronic database$ or bibliographic database$ or computeri?ed database$ or online database$).tw,sh.
8. (pooling or pooled or mantel haenszel).tw,sh.
9. (peto or dersimonian or der simonian or fixed effect).tw,sh.
10. (retraction of publication or retracted publication).pt.
11. 1 and 11 meta-analysis.pt.
12. meta-analysis.sh.
13. (meta-analys$ or meta analys$ or metaanalys$).tw,sh.
14. (systematic$ adj5 review$).tw,sh.
15. (systematic$ adj5 overview$).tw,sh.
16. (quantitativ$ adj5 review$).tw,sh.
17. (quantitativ$ adj5 overview$).tw,sh.
18. (quantitativ$ adj5 synthesis$).tw,sh.
19. (methodologic$ adj5 review$).tw,sh.
20. (methodologic$ adj5 overview$).tw,sh.
21. (integrative research review$ or research integration).tw.
22. or/13-23
23. 12 or 24

Older people search

24. Aged/
25. Exp frail elderly/
26. Exp health services for the aged/
27. Geriatrics/
28. (Frail or frailty or vulnerable or vulnerability or weak or infirm or elder$ or geriatric$).tw
29. Exp Aging, Premature/
30. Exp Mobility Limitation/
31. older adult$.tw.
32. 26 or 27 or 28 or 29 or 30 or 31 or 32 or 33

Discharge search

33. ((patient$ adj2 discharg$) or (hospital$ adj2 discharg$) or (discharg$ adj2 plan$) or (supported adj2 discharg$)).tw.
34. ((discharge or Re$ablement or rehab$) adj2 (plan$ or service$ or program$ or intervention$ or model or pattern or occupational therap$ or physiotherap$ or physical therap$)).tw
35. ((organi?ed or multidisciplinary) adj2 (discharge adj2 team$)).tw.
36. (length adj2 stay).tw.
37. (Community adj1 (based or care)).tw.
38. ((service or care$) adj2 (provis$ or provid$ or pattern$ or access$ or delivery or model$)).tw
39. ((home or home?based or home nursing or community or post?discharge or house) adj2 (rehabilitation or support$ or care or visit)).tw
40. hospital adj2 home.tw
41. Exp continuity of Patient Care [MH]
42. Exp Residential Facilities [MH]
43. Exp Community Health Services [MH]

44. (home adj1 (based or care)).tw.
45. 35 or 36 or 37 or 38 or 39 or 40 or 41 or 42 or 43 or 44 or 45 or 46 or 47

Combined searches in Medline
46. 25 and 34 and 47
47. limit 48 to human
48. limit 49 to English language

Review search is from http://clinicalevidence.bmj.com/x/set/static/ebm/learn/665076.html *- identified from Lee et al 2012 [31]*
